# Influence of Phosphate Marinades on the Quality and Flavor Characteristics of Prepared Beef

**DOI:** 10.3390/molecules30010202

**Published:** 2025-01-06

**Authors:** Wanqi Wang, Maomao Zeng, Qiuming Chen, Zhaojun Wang, Zhiyong He, Jie Chen

**Affiliations:** 1 State Key Laboratory of Food Science and Resources, Jiangnan University, Wuxi 214122, China; wwq735487234@163.com (W.W.); mmzeng@jiangnan.edu.cn (M.Z.); chenqm@jiangnan.edu.cn (Q.C.); zhaojun.wang@jiangnan.edu.cn (Z.W.); zyhe@jiangnan.edu.cn (Z.H.); 2 School of Food Science and Technology, Jiangnan University, Wuxi 214122, China

**Keywords:** phosphates, prepared beef, beef quality, flavor profile, lipid oxidation

## Abstract

Phosphate has been widely used in beef to improve processing characteristics such as tenderness and water-holding capacity. However, the effects of phosphates on the quality and especially the flavor of beef are not well understood. This study investigated the influence of eight different phosphate marinade solutions on the quality and flavor of prepared beef. The results revealed that the thawing loss in the control group was 11.47%, and NaCl with sodium hexametaphosphate (SYCP) had the lowest thawing loss, with a value of 2.13%, which was reduced by 81.43% as compared to the control group. The shear force of the control group was 3.85 kg, and the shear work was 10.03 kg. The best tenderness was recorded in the NaCl with sodium hexametaphosphate (SYST) group, which had a shear force of 1.14 kg and shear work of 3.34 kg. The incorporation of phosphates suppressed fat oxidation and increased the total free amino acid content. Additionally, the levels of certain key volatile flavor compounds, particularly those associated with fat oxidation, such as hexanal, heptanal, octanal, and nonanal, were reduced. In terms of sensory evaluation, juiciness, flavor, tenderness, and overall acceptability in the treatment group were significantly increased (*p* < 0.05). Overall, the results indicate that adding phosphates can enhance the quality of processed beef, inhibit lipid oxidation, and improve sensory evaluation.

## 1. Introduction

Prepared beef products are non-ready-to-eat meat items that undergo various processing, including cutting, salting, rolling, and seasoning. These products are stored, transported, and sold at low temperatures before a second cooking or processing phase prior to consumption [1]. With its convenience, prepared meat is suitable for the current fast-paced lifestyles and the demand for high-quality food options. It is projected that global average meat consumption may be up to 90 kg by 2050 [2]. Among the attributes affecting consumer satisfaction, beef tenderness has historically been considered as the most important characteristic. However, as consumer expectations evolve, the flavor of beef has become a key determinant influencing purchasing decisions [3]. A study on European beef samples revealed that flavor preference accounted for the largest proportion of beef palatability (39%), followed by tenderness (31%) and juiciness (24%) [4]. Flavor plays a significant role in driving beef demand and distinguishes it from other protein sources. Therefore, understanding the flavor of prepared beef while maintaining tenderness is essential.

Current methods for improving meat tenderness mainly include physical, biological, and chemical approaches. One of the most common chemical methods involves using phosphates to improve beef texture. Research indicates that adding 0.5% sodium tripolyphosphate can significantly stabilize prepared roast beef and reduce oxidation [5]. Additionally, phosphate has been shown to significantly improve beef tenderness and juiciness [6]. The palatability and color stability of beef biceps femoris can also be enhanced through the use of phosphates and sodium chloride [7]. Most existing studies on phosphates in meat products focus on their effects on water retention, emulsification stability, cooking yield, and sensory attributes [8,9]. However, the impact of different phosphate types and concentrations on beef flavor components remains unclear. Changes in certain flavor components can significantly affect consumer preferences, while others may have minimal effects. Thus, identifying the key ingredients that contribute to beef flavor is necessary.

Recently, gas chromatography–mass spectrometry (GC-MS) has attracted attention as a method for analyzing meat flavor due to its rapid, straightforward, and non-destructive nature. It is widely used for flavor detection, spoilage monitoring, and differentiating meat types [10]. Headspace solid-phase microextraction (HS-SPME) is a common technique for analyzing volatile organic compounds in food, offering benefits such as simplicity, speed, and solvent-free extraction [11,12]. HS-SPME has been used to analyze the volatile flavor compounds of bamboo shoots under varying heat treatment conditions [13], as well as different beef cuts [14]. Additionally, HS-SPME-GC-MS has been also used to identify volatile flavor compounds in crayfish meat, revealing 14 volatile substances as characteristic aroma compounds [15]. However, research on the flavor of prepared beef after the addition of phosphates is limited, and the effects of phosphates on beef flavor and their changing characteristics remain poorly understood.

This study investigated the quality characteristics and flavor of prepared beef injected with eight different phosphate marinades. The impact of various phosphate types on beef flavor production was assessed using HS-SPME-GC-MS while ensuring quality. In addition, potential reasons for these flavor changes were explored.

## 2. Results

### 2.1. Thawing Loss, pH, and Color

**Figure 1:**
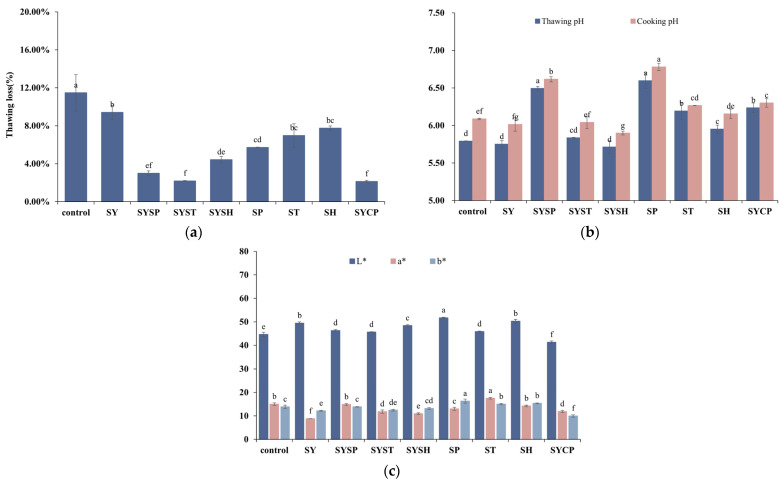
The values of different phosphates on: (**a**) Thawing loss; (**b**) pH; (**c**) Color. ^a–g^ indicate a significant difference between different groups at *p* < 0.05 (*n* = 9).

Thawing loss is closely related to meat tenderness and quality, serving as an indicator of water-holding capacity in meat products. A higher thawing loss reflects a reduced water-holding capacity. The effects of different phosphate treatments on thawing loss in prepared beef are shown in Figure 1a. Different phosphate treatments significantly influenced the thawing loss in beef (*p* < 0.05). The control group showed a thawing loss of 11.47%, while treatment groups ranged between 2.13% and 9.41%, all showing significantly lower thawing loss compared to the control (*p* < 0.05). This decrease is likely due to the interaction between phosphates and metal ions, which promotes actin dissociation and increases meat protein solubility, thereby improving water-holding capacity and reducing thawing loss [16]. Among treatments, the SYSP, SYST, SYSH, and SYCP groups showed the most notable reductions in thawing loss, with SYCP achieving the lowest value, reducing thawing loss by 81.43% compared to the control. This effect may be due to the combined impact of salt and phosphate on significantly enhancing meat water-holding capacity [17].

The pH values of thawed and cooked meat are shown in Figure 1b. The thawed pH of the control group was 5.79, while the pH in the SYSP, SP, ST, SH, and SYCP groups was significantly higher (*p* < 0.05), ranging from 5.95 to 6.60, with the SP group showing the highest pH of 6.60, likely due to the mild basicity of the phosphate solution. There was no significant difference (*p* > 0.05) in pH between the SY, SYST, and SYSH groups and the control, which may be attributed to the neutral pH of the NaCl treatment. After cooking, the pH values followed a similar trend to that observed post-thawing. The increase in pH in samples with salt and phosphate after cooking may result from the disruption of chemical bonds (hydrogen bonding, hydrophobic interactions, etc.) in proteins upon heating, which reduces acidic groups on the protein or encapsulates them, thereby increasing the pH [18].

Meat color, which affects consumer perceptions of freshness and quality, was assessed via lightness (L*), redness (a*), and yellowness (b*). The L* value of the control group was 44.64, while treated samples showed higher L* values (Figure 1c). This may result from phosphate treatments increasing the ionic strength, enhancing water-holding capacity, and subsequently making the sample appear brighter due to surface moisture refracting more light. The b* values of SP, ST, and SH groups exceeded those of the control, probably due to phosphate-induced pH modulation that impacted beef color [19].

### 2.2. Tenderness

**Figure 2:**
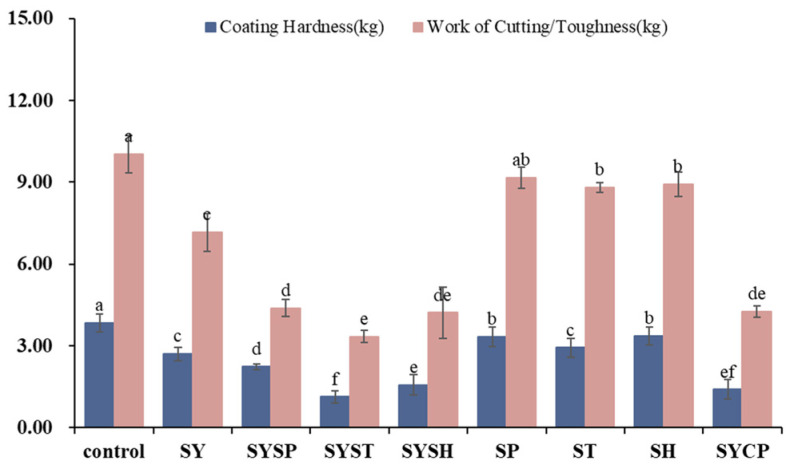
The values of different phosphates on the tenderness of prepared beef. ^a–f^ indicate a significant difference between different groups at *p* < 0.05 (*n* = 9).

Shear force is inversely proportional to beef tenderness, and the effect of different phosphate treatments on beef tenderness is shown in Figure 2. The control group had a shear force of 3.85 kg and a shear work of 10.03 kg. All treatment groups demonstrated significantly lower (*p* < 0.05) shear force and work compared to the control, with values ranging from 1.14 to 3.36 kg and 3.34 to 9.16 kg, respectively. This reduction may result from phosphates dissociating actomyosin into actin and myosin, reducing muscle fiber cross-linking [20]. Among the treatment groups, SYSP, SYST, and SYSH exhibited significantly lower shear force and shear work (*p* < 0.05) than the SP, ST, and SH groups, with the SYST group achieving the lowest values, showing reductions of 70.39% and 66.70% compared to the control. The combination of phosphate and sodium chloride appears to enhance protein solubility, water-holding capacity, and overall meat softness and juiciness [17].

Table 1 shows the effect of different phosphates on beef texture. The control hardness was 3884.79 g, whereas treated groups ranged between 2311.33 and 3633.10 g, all significantly lower than the control (*p* < 0.05). Hardness was especially reduced in the SYSP, SYST, SYSH, and SYCP groups, with these groups showing highly significant differences. Springiness in the control was 0.60, with all treatment groups showing higher values. This increase was significant (*p* < 0.05) in the SYSP, SYST, SYSH, and SYCP groups, though not significant (*p* > 0.05) in the other groups. Trends in chewiness and hardness mirrored these findings, with the control chewiness at a maximum of 1617.24, while all treatment groups showed lower chewiness, indicating improved tenderness. Decreased hardness and chewiness alongside increased springiness are likely due to phosphate-induced actomyosin dissociation and enhanced water-holding capacity, which collectively improve tenderness and texture [21]. No significant differences (*p* > 0.05) were observed in resilience values across groups, with resilience results aligning with shear force reduction due to phosphate effects.

### 2.3. Free Amino Acid Content

Amino acids contribute essential taste attributes to beef, with different amino acids imparting distinct flavors. For example, umami amino acids include aspartic acid (Asp) and glutamic acid (Glu); sweet amino acids include serine (Ser), glycine (Gly), threonine (Thr), alanine (Ala), proline (Pro), and lysine (Lys); bitter amino acids include tyrosine (Tyr), valine (Val), isoleucine (Ile), leucine (Leu), histidine (His), and arginine (Arg); and tyrosine (Tyr) and phenylalanine (Phe) contribute aromatic characteristics [22]. The free amino acid content across treatment groups is summarized in Table 2. In the control group, the total free amino acid content was 0.84 g/100 g, which varied following phosphate treatment. However, differences in free amino acid content were not significant (*p* > 0.05) between SYST, ST, and SH groups and the control. In contrast, the other treatment groups displayed significantly higher free amino acid content (*p* < 0.05) than the control. This increase may be attributed to the effect of phosphate on myofibrillar proteins’ solubility, which can promote the release of endogenous proteases and the enzymatic cleavage of proteins, resulting in the release of additional small molecules and a corresponding rise in free amino acid content [23].

### 2.4. TBARS Values

**Figure 3:**
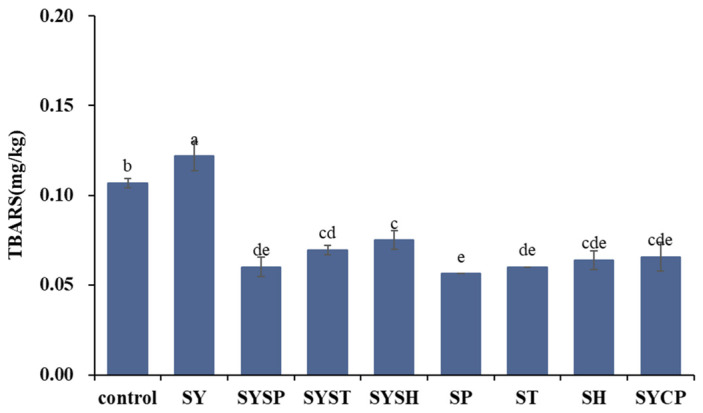
The values of different phosphates on TBARS values of prepared beef. ^a–e^ indicate a significant difference between different groups at *p* < 0.05 (*n* = 9).

Under high-temperature conditions, malondialdehyde, a byproduct of lipid oxidation, reacts with thiobarbituric acid (TBA) to form a reddish-brown substance, which has an absorption at 532 nm. The intensity of this absorption correlates linearly with malondialdehyde concentration, allowing the TBA reactive substance (TBARS) value to serve as an indicator of lipid oxidation, where higher values indicate greater oxidation. As shown in Figure 3, the TBARS value in the control group was 0.1068 mg/kg. In contrast, the SY group had a significantly higher value of 0.1218 mg/kg (*p* < 0.05), which may be due to the oxidation effect of added salt on lipids. TBARS values for other treatment groups ranged from 0.0562 to 0.0749 mg/kg, all significantly lower than the control (*p* < 0.05), indicating that phosphate addition effectively inhibited lipid oxidation in beef. This may be attributed to the ability of phosphates to chelate metal ions, thereby reducing metal-catalyzed oxidation and helping to preserve beef quality [24]. Phosphate addition has been reported to lower free fatty acid levels, and TBARS values could be reduced by the addition of phosphates, indicating that phosphate delays lipid hydrolysis and reduces the oxidation degree [25]. Lipid oxidation is closely correlated with the flavor compound formation during the processing and storage of beef, and thus, the addition of phosphate may have an effect on the flavor quality of prepared beef.

### 2.5. Volatile Flavor Compounds

**Figure 4:**
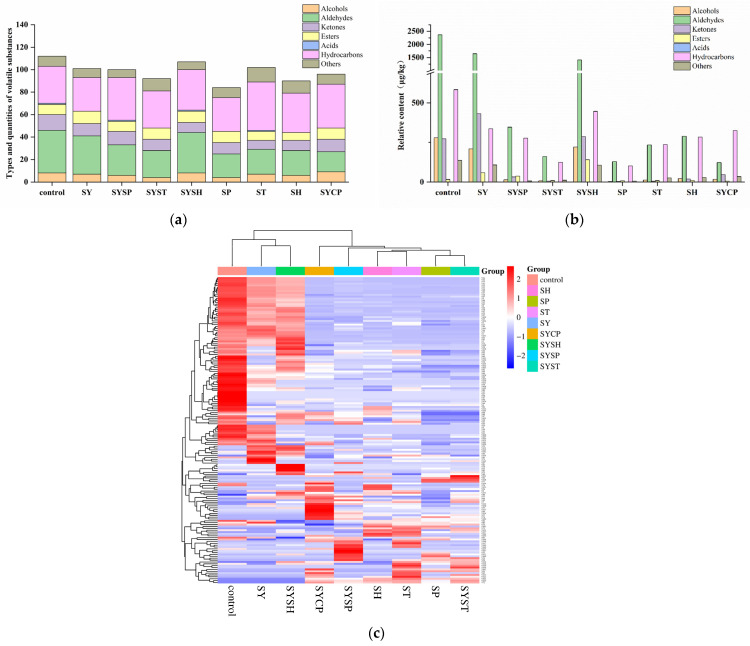
The comprehensive analysis of volatile flavor compounds in prepared beef: (**a**) types and quantities of volatile substances; (**b**) relative content; (**c**) heatmap of flavor compounds.

The volatile flavor compounds of beef samples from different treatment groups were analyzed and identified, resulting in a total of 164 compounds categorized into seven classes, designated as V1 to V164. These classes include alcohols (10), aldehydes (45), ketones (22), esters (15), acids (1), hydrocarbons (57), and others (14). The categories and quantities of volatile compounds across treatment groups are shown in Figure 4a. In the control group, 112 volatile compounds were detected, with treatment groups showing reductions in the number of detected compounds, ranging from 84 to 107. Compared to the control, all treatment groups showed a decrease in alcohols, aldehydes and ketones. Additionally, ester content increased in the SY, SYST, SYSH, SP and SYCP groups; hydrocarbons decreased in the SY and SP groups but increased in other treatment groups; while other compounds increased in the SYST, ST and SH groups. In Figure 4b, total volatile content in the control was 3659.80 μg/kg, while treatment groups exhibited reductions, with the SP group exhibiting the largest decrease to 255.62 μg/kg, and the SY group retaining a moderate reduction to 2789.10 μg/kg. Relative to the control, alcohols, aldehydes, hydrocarbons, acids and other classes decreased significantly (*p* < 0.05) in all treatment groups, while ketones increased only in the SY group, and ester content was notably higher in the SYSH group. To clearly see the volatile differences between the samples in the different treatment groups, visualize the GC-MS results using the heat map in Figure 4c, where the color change of the spots from blue to red indicates an increase in the amount of the compound. The results showed that the content of aldehydes, alcohols and ketones in the control group was relatively high, while the content of these compounds was relatively reduced in the treatment group. Although the content of some aldehydes, alcohols and ketones in the treatment group was reduced, the formation of some new substances was also observed, which made a significant difference between the treatment group and the control group. The above results further support the quantitative data shown in Figure 4a,b.

Aldehydes, mainly derived from lipid oxidation and amino acid degradation, are key contributors to beef aroma due to their low odor thresholds, particularly small-molecule branched aldehydes. In this study, 45 aldehydes were detected, with hexanal, octanal, heptanal, pentanal, and nonanal present at higher levels, primarily originating from unsaturated fatty acid degradation. Within the volatile compounds derived from lipid oxidation, aldehydes are the most abundant [26]. Aldehyde content in the treated groups were significantly lower than in the control (*p* < 0.05), This may be due to the fact that the degree of lipid oxidation in the treatment group is significantly lower than that in the control group.

Alcohols, which typically have fruity and floral notes, were found in 10 different forms. Their high odor threshold suggests they contribute minimally to beef aroma. The treatment groups showed significantly lower alcohol content than the control (*p* < 0.05), with 1-octene-3-ol, 1-pentanol, 1-hexenol and 1-octanol present at higher levels in the control group. The contents of 1-hexanol and 1-pentanol in the treatment group were decreased. 1-pentanol was derived from the degradation of lipid hydroperoxide, and 1-hexanol was derived from the secondary product of linoleic acid oxidation [27]. This may be due to the lower peroxides produced in the treatment group than in the control group.

Ketones, generally derived from amino acid degradation, Maillard reaction, and lipid thermal oxidation, totaled 24 in this study. The control had higher levels of 2,3-butanedione, contributing a sweet and creamy aroma, while 2,3-octanedione was unique to the treated groups. Certain ketones act as intermediates in heterocyclic compound formation [28], which may slightly enhance beef flavor.

A diverse range of hydrocarbons, totaling 60, were detected, with both saturated and unsaturated forms. The types of hydrocarbons increased in the SYSP, SYST, SYSH, ST, SH, and SYCP groups compared to the control. Previous studies indicated hydrocarbons contributed minimally to aroma perception due to high odor thresholds [29]. Esters, which impart sweet and fruity flavors and aromas, are formed by esterification between alcohols and fatty acids [30]. A total of 15 esters were detected, though with low overall content and diversity, as low volatility and minimal modulation effects likely limit their role in flavor [31]. Other compounds detected were at lower concentrations and similarly had minimal aroma impact due to high odor thresholds.

### 2.6. Odor Activity Values (OAVs)

The role of volatile flavor compounds in aroma is influenced not only by their concentration, but also by their OAV [32]. Compounds with OAV > 1 are considered key odor-active compounds and have a significant impact on aroma. A higher OAV indicates a greater contribution to the aroma. Compounds with OAVs between 0.1 and 1 contribute less to the overall aroma [33].

The OAV values of volatile flavor compounds in beef subjected to different treatments are shown in Table 3. According to the odor thresholds of these compounds, we identified 24 substances, including 1 alcohol, 15 aldehydes, 4 ketones, 1 hydrocarbon, 1 furan, and 2 sulfides. Compounds such as 2,4-decadienal (fat, fried) and hexanal (grass, butter, fat), heptanal (grass, butter, fat), 2-nonenal (fat, fried, fat, green), 1-octen-3-ol (moldy, mushrooms), (E,E)-2,4-nonadienal (fat, fried, green), and (E)-2-octenal (green, floral) are produced from the autoxidation of ω-6 fatty acids (e.g., linoleate and arachidonic acid). Similarly, (Z)-2-decenal (fat, fried), decanal (fat, waxy), octanal (fat, waxy), and nonanal (fat, waxy) originated from the autoxidation of ω-9 fatty acids (e.g., oleic acid). Compounds derived from ω-3 fatty acids (e.g., linolenic acid), such as (E,E)-2,4-heptadienal (fat, green) and 3,5-octadien-2-one (fruity, fat), also contributed to the aroma profile [34,35]. Most of these compounds have pleasant aromas, such as sweet, fresh, fruit, vegetable, and floral aromas, and can enhance the flavor characteristics of meat products.

In comparison to the control group, the OAV values of compounds in the phosphate treatment group decreased, indicating a reduction in the beef-specific aroma due to the phosphate treatment. These compounds have also been detected in other cooked foods, including lamb and foals [36,37,38]. The hydrocarbon d-limonene (citrus, lemon) existed in all groups and has been identified as a key aroma component in cooked beef meatballs during storage [39]. These terpenoids may interact with other compounds derived from chemical reactions, shaping the flavor profile of beef [40].

Reports have identified 2-pentylfuran (green beans, butter) as an odor-active compound in meat products, contributing to the overall flavor profile [41,42,43]. Sulfur-containing compounds such as dimethyl disulfide (mold, rubber, onion) and dimethyl trisulfide (potato, vegetables) have a low threshold and intense flavors, known to originate from sulfur amino acids, thiamine, or glutathione [44]. Compared to the control group, the SYSH group exhibited minimal alterations in the overall odor-active compound profile. However, it significantly suppressed the formation of volatile compounds associated with lipid oxidation, particularly hexanal, octanal, and nonanal. This indicates that SYSH treatment can help maintain flavor stability by limiting undesirable oxidation-related off-flavors. In contrast, while the number of odor-active compounds in the SYST, ST, and SH groups decreased, the OAV value of dimethyl disulfide was significantly higher in these treated groups. This suggests a reduction in lipid-oxidation-related products, with Maillard reaction products becoming the dominant contributors to the volatile profile. Consequently, this shift influences the generation pathways and distribution of flavor compounds. It was speculated that the incorporation of phosphates effectively inhibits lipid oxidation, thereby reducing the generation of off-flavors while promoting the formation of certain Maillard reaction products, such as sulfur-containing compounds, which can further enhance the overall flavor profile of meat products.

### 2.7. Sensory Evaluation

Table 4 shows the effects of the addition of different types of phosphates on the sensory properties of prepared beef. Compared with the control group, the addition of phosphate significantly improved the juiciness, flavor, tenderness, and overall acceptability of meat (*p* < 0.05), and the improvements in juiciness and tenderness were consistent with the results of thawing loss and texture properties, which further verified the effect of phosphate on the water retention ability of meat products. However, there were no significant differences in appearance (*p* > 0.05), which may be due to the small changes in muscle color caused by phosphate. The combination of juiciness, flavor, and tenderness has resulted in a significant increase in overall consumer acceptance of phosphate-treated beef. Overall, the addition of phosphate improved the sensory quality of beef.

## 3. Materials and Methods

### 3.1. Materials and Chemicals

Beef tenderloin was purchased from Jiangsu Niu Nair International Trade Co., Ltd. (Nantong, China) and transported under cold-chain conditions. Sodium pyrophosphate was purchased from Guizhou Wengfu Jianfeng Chemical Co., Ltd. (Guiyang, China). Sodium tripolyphosphate and sodium hexametaphosphate were purchased from Hubei Xingfa Chemical Group Co., Ltd. (Yichang, China). Salt was purchased from Jiangsu Salt Industry Group Co., Ltd. (Nanjing, China). Flavor standards were purchased from J&K Scientific (Beijing, China).

### 3.2. Sample Preparation

The meat was cut into 27 steaks (8 cm × 8 cm × 2 cm, 200 g each) after removing external fat and connective tissue. The steaks were divided into 9 groups (*n* = 3 per group), each prepared with different phosphate solutions: (1) control, (2) 1.5% NaCl (SY), (3) 1.5% NaCl + 0.5% sodium pyrophosphate (SYSP), (4) 1.5% NaCl + 0.5% sodium tripolyphosphate (SYST), (5) 1.5% NaCl + 0.5% sodium hexametaphosphate (SYSH), (6) 0.5% sodium pyrophosphate (SP), (7) 0.5% sodium tripolyphosphate (ST), (8) 0.5% sodium hexametaphosphate (SH), (9) 1.5% NaCl with a composite sodium phosphate mix (sodium pyrophosphate/sodium tripolyphosphate/sodium hexametaphosphate = 2:2:1) (SYCP). Phosphate solutions were injected through an injection machine (IS400-171 Inject Star, Europe) with a water injection rate of 20% of the meat weight, followed by vacuum tumbling (0.06‒0.08 MPa) at 9 rpm 8 °C for 1 h. Samples were kept at 4 °C for 24 h, vacuum-packaged, and frozen at ‒20 °C for 3 days and analyzed after thawing at 4 °C for 24 h.

### 3.3. Thawing Loss

After thawing at 4 °C for 24 h, each sample was blotted dry, and weights were recorded before (*m*1) and after (*m*2) thawing. Thawing loss (%) was calculated using the following equation:

(1) (m1−m2)/m1×100%

### 3.4. pH and Color Measurement

The pH was determined following a modified protocol [45]. Each 10 g sample was homogenized in 20 mL distilled water (IKA T18 digital UltraTurrax, Staufen, Germany) at 1000 rpm for 15 s, and the pH values were measured using a pH meter (FE-20, Mettler-Toledo, Shanghai, China). Color (L*, a*, b* values) was measured using an automatic colorimeter (WB2000-IXA, Kangguang Optical, Beijing, China).

### 3.5. Textural Properties

Textural properties were assessed with a texture analyzer (TA-XT plus, Stable Micro Systems, Vienna Court, Lammas Road, Godalming, UK). Shear force was measured on 10 mm cubes of cooked meat under 100% strain with speeds of 1.5 mm/s (before and during) and 10 mm/s (after testing) using an A/CKB probe. At least ten replicates were carried out per sample. For texture profile analysis (TPA), hardness, springiness, chewiness, and resilience were assessed using a P/36R probe, with the strain set at 50%. The crosshead speed was set at 2 mm/s before, 1 mm/s during, and 2 mm/s after compression, with a 5 s interval between two cycles. The crosshead speed was set to 1 mm/s before, 1 mm/s during, and 2 mm/s after the second compression. At least ten replicates were carried out per sample.

### 3.6. Determination of Free Amino Acids by HPLC

Free amino acids were extracted and identified following the method of [46]. First, a 1.0 g cooked beef mince sample was ultrasonically extracted in 10 mL of 0.1 mol/L HCl and centrifuged at 15,000× *g* for 15 min. The supernatant underwent precolumn derivatization and HPLC analysis. For derivatization, 10 μL of the supernatant was mixed with 5 mg/mL of o-phthalaldehyde and 10 mg/mL of 9-fluorenylmethyl chloroformate in the autosampler for 20 s before injection. Amino acids were separated and detected on an Agilent 1200 HPLC system (Santa Clara, CA, USA) equipped with a G1314A variable wavelength detector and an ODS HYPERSIL column (4.6 mm × 250 mm id, 5 μm). Solvent A was 27.6 mmol/L sodium acetate–methanol–tetrahydrofuran (500:0.11:2.5, *v*/*v*/*v*, pH 7.2), and solvent B was 80.9 mmol/L sodium acetate–methanol–acetonitrile (1:2:2, *v*/*v*/*v*). Gradient elution was applied at a flow rate of 1 mL/min as follows: 0–0.1 min at 8% B; 0.1–17 min, 8–50% B; 17–20.1 min, 50–100% B; and 20.1–24 min, 100–0% B. The column temperature was 40 °C, the injection volume was 10 μL, and the detector wavelength was set to 338 nm. Data acquisition and processing were performed using Agilent ChemStation software, with amino acid standards used for quantification.

### 3.7. Determination of TBARS Values

The fat oxidation indices were determined according to a modified method of [47]. Five grams of ground, raw beef was mixed with 20 mL of 7.5% trichloroacetic acid (TCA) containing 0.1% EDTA, shaken for 30 min, and filtered. To 5 mL of filtrate, 5 mL of 0.02 mol/L TBA solution was added and incubated at 90 °C for 40 min. After cooling for 1 h, the sample was centrifuged at 2000 rpm for 5 min. The supernatant was mixed with 5 mL of chloroform and layered, and the upper layer was measured at 532 nm and 600 nm. The TBARS value (*T*, mg/kg) was calculated as follows:

(2) T=(A532−A600)×4.68÷(m×5/50)

where *m* is the mass of the sample (g), 4.68 is the conversion factor (mg malondialdehyde/OD value), 5 is the filtrate volume used in the assay, and 50 is the initial sample volume in TCA solution.

### 3.8. Volatile Compounds Using HS/SPME-GC/MS

To extract volatile compounds, 2.0 g of cooked beef sample was added into a 20 mL vial with 2 μL of 2-methyl-3-heptanone (118.2 μg/mL) as an internal standard and then sealed. A conditioned SPME fiber (50/30 μm DVB/CAR/PDMS) was used to extract volatile flavor components at 60 °C for 30 min. The fiber was then inserted into the injection port for 7 min for desorption and separation.

GC conditions: A DB-WAX capillary column (30 m × 0.25 mm × 0.25 μm) with helium as the carrier gas (1 mL/min flow rate) was used. The detector and inlet temperatures were both set to 250 °C. The column temperature was programmed as follows: 40 °C for 3 min, ramped to 100 °C at 3–13 min, then to 250 °C from 13 to 28 min, held at 250 °C for 5 min. Injection was performed in splitless mode. MS parameters were as follows: electron impact ionization (EI) was used at 70 eV, with a mass scan range of 35‒450 *m/z*. The ion source and inlet temperature were maintained at 250 °C. Volatile compounds were identified using the Wiley standard mass spectra library (NIST 17 and 17 s), the retention index (RI), and literature data. RI values were calculated using C8‒C26 n-alkanes and compared with the RI values of the flavor compounds from NIST17 and NIST17s databases and the literature for compound identification (Supplementary Materials).

### 3.9. Odor Activity Value (OAV) Calculation

Odor activity values (OAVs) were calculated to evaluate the influence of aroma-active compounds on overall odor by dividing the concentration of a compound by its odor threshold. The formula used to obtain the OAV of each volatile flavor compound was OAVi = Ci/Ti, where Ci stands for the content of compound i (μg/kg) and Ti for its olfactory threshold (μg/kg). Compounds with OAV ≥ 1 were considered contributors to aroma, with contributions proportional to their OAV values.

### 3.10. Sensory Evaluation

Sensory analysis of beef was conducted immediately after preparation. Twenty trained panelists (9 females and 11 males, aged 20–35 years) evaluated the sensory attributes of cooked meat using a 9-point hedonic scale. All sensory tests were conducted under white illumination at 25 °C and 50–70% relative humidity. The research protocol for the sensory group was reviewed and approved by the Ethics Committee of the Medical School of Jiangnan University with the reference number (JNU202412RB002), complying with the ethical standards in human research. The evaluators were all trained and were familiar with the method to evaluate cooked beef. The data in this study were collected with the informed consent of all evaluators. All evaluators agreed to take part and use their information, and were fully informed how to use the data in this study. Approximately 8 g of each sample was served to panelists, who assessed the appearance, juiciness, flavor, tenderness, and overall acceptability of the coded samples. Each sample was labeled with a unique three-digit random code and presented in random order. Scores ranged from 1 (extremely dislike) to 9 (extremely like), and the mean values of the scores from all twenty panelists were calculated for each sample and used in the data analysis.

### 3.11. Statistical Analysis

All experiments were conducted in triplicate, and results were presented as mean ± standard deviation. To describe the significance of the main effects (*p* < 0.05), a one-way analysis of variance (ANOVA) along with a Duncan’s multiple comparison was assessed using IBM SPSS Statistics 23 software (IBM, Armonk, NY, USA).

## 4. Conclusions

The addition of phosphates effectively improved the water-holding capacity and texture characteristics of beef, delayed lipid hydrolysis, and reduced the extent of oxidation in prepared beef. Meanwhile, in terms of sensory evaluation, the juiciness, flavor, tenderness and overall acceptability of beef were improved. Phosphates significantly influenced the odor of the beef products. Analysis indicated that this effect was mainly due to the suppression of key volatile flavor compounds with an OAV greater than 1 in the treatment groups. Therefore, it can be concluded that the incorporation of phosphates can enhance both the quality and overall flavor profile of prepared beef. In particular, the four treatment groups of SYSP, SYST, SYSH, and SYCP were most effective in improving the water holding capacity, texture, flavor, and overall sensory acceptability of beef products, showing the best overall performance.

## Figures and Tables

**Table 1:** Values in TPA of prepared beef based on the addition of varying types of phosphate *.

Group	Hardness (N)	Springiness (cm)	Chewiness (N × cm)	Resilience (-)
control	38.07 ± 3.56 ^a^	0.60 ± 0.05 ^b^	15.85 ± 2.57 ^a^	0.25 ± 0.01 ^bc^
SY	35.60 ± 3.33 ^ab^	0.65 ± 0.03 ^b^	14.53 ± 2.03 ^ab^	0.25 ± 0.03 ^abc^
SYSP	22.65 ± 5.22 ^e^	0.78 ± 0.04 ^a^	11.27 ± 1.72 ^cd^	0.25 ± 0.03 ^bc^
SYST	27.30 ± 2.90 ^de^	0.78 ± 0.04 ^a^	12.81 ± 1.41 ^bc^	0.26 ± 0.01 ^ab^
SYSH	22.77 ± 2.30 ^e^	0.78 ± 0.05 ^a^	9.73 ± 1.36 ^d^	0.27 ± 0.02 ^a^
SP	33.00 ± 1.13 ^bc^	0.60 ± 0.06 ^b^	14.13 ± 1.86 ^ab^	0.22 ± 0.01 ^de^
ST	32.85 ± 1.69 ^bc^	0.62 ± 0.06 ^b^	13.69 ± 1.40 ^abc^	0.22 ± 0.01 ^de^
SH	29.98 ± 1.57 ^cd^	0.62 ± 0.04 ^b^	13.02 ± 1.59 ^bc^	0.21 ± 0.02 ^e^
SYCP	27.22 ± 0.35 ^de^	0.75 ± 0.03 ^a^	12.52 ± 1.44 ^bc^	0.23 ± 0.02 ^cd^

* Different superscript letters in the same column represent a significant difference (*p* < 0.05).

**Table 2:** Values in free amino acid content of prepared beef based on the addition of varying types of phosphate *.

Species	Content (mg/100g)								
	Control	SY	SYSP	SYST	SYSH	SP	ST	SH	SYCP
asp	40.1 ± 7.49 ^a^	9.70 ± 0.30 ^b^	10.1 ± 0.43 ^b^	7.95 ± 1.15 ^b^	7.25 ± 0.16 ^b^	8.77 ± 0.37 ^b^	9.39 ± 0.24 ^b^	7.08 ± 1.79 ^b^	8.33 ± 1.88 ^b^
glu	65.8 ± 0.63 ^cd^	50.0 ± 2.79 ^e^	80.2 ± 8.29 ^ab^	57.8 ± 2.35 ^de^	51.2 ± 5.01 ^e^	72.2 ± 7.54 ^bc^	92.5 ± 9.33 ^a^	48.0 ± 4.14 ^e^	57.6 ± 2.83 ^de^
ser	44.1 ± 1.29 ^a^	27.3 ± 0.98 ^cd^	34.2 ± 3.97 ^bc^	31.9 ± 0.75 ^bc^	26.1 ± 0.18 ^cd^	27.3 ± 0.84 ^cd^	28.9 ± 4.63 ^cd^	23.1 ± 0.83 ^d^	38.0 ± 7.64 ^ab^
his	24.0 ± 4.28 ^a^	18.5 ± 1.36 ^a^	17.0 ± 6.28 ^a^	12.0 ± 1.02 ^a^	11.4 ± 0.80 ^a^	14.2 ± 0.66 ^a^	11.3 ± 14.1 ^a^	11.5 ± 2.52 ^a^	16.3 ± 2.81 ^a^
gly	41.4 ± 0.23 ^bc^	41.4 ± 0.15 ^bc^	37.7 ± 3.14 ^d^	38.5 ± 1.22 ^cd^	42.0 ± 0.35 ^b^	42.2 ± 0.79 ^b^	34.2 ± 0.99 ^e^	46.8 ± 1.34 ^a^	38.5 ± 0.93 ^cd^
thr	41.8 ± 0.83 ^a^	31.8 ± 0.49 ^bc^	35.0 ± 4.43 ^b^	30.5 ± 1.67 ^bc^	28.0 ± 0.92 ^c^	30.8 ± 0.60 ^bc^	27.4 ± 3.13 ^c^	26.5 ± 1.49 ^c^	30.8 ± 1.82 ^bc^
arg	43.1 ± 0.51 ^c^	42.9 ± 4.04 ^c^	62.8 ± 0.58 ^a^	50.2 ± 0.32 ^b^	40.6 ± 0.81 ^c^	44.4 ± 2.14 ^c^	58.2 ± 1.40 ^a^	44.1 ± 3.27 ^c^	49.9 ± 1.54 ^b^
ala	344 ± 0.70 ^f^	485 ± 5.26 ^b^	524 ± 4.79 ^a^	364 ± 5.11 ^e^	493 ± 5.19 ^b^	485 ± 7.11 ^b^	372 ± 1.83 ^e^	461 ± 7.95 ^c^	446 ± 0.25 ^de^
tyr	24.2 ± 0.95 ^b^	34.5 ± 3.65 ^ab^	36.0 ± 13.0 ^ab^	33.8 ± 6.83 ^ab^	35.1 ± 5.67 ^ab^	33.8 ± 6.44 ^ab^	30.7 ± 0.50 ^ab^	33.6 ± 0.48 ^ab^	42.8 ± 13.2 ^a^
cys-s	0.76 ± 0.43 ^a^	0.66 ± 0.35 ^a^	0.51 ± 0.09 ^a^	0.43 ± 0.03 ^a^	0.51 ± 0.19 ^a^	0.69 ± 0.42 ^a^	0.35 ± 0.14 ^a^	0.44 ± 0.03 ^a^	0.37 ± 0.24 ^a^
val	31.2 ± 0.21 ^a^	37.4 ± 2.27 ^a^	36.8 ± 0.13 ^a^	29.4 ± 2.05 ^a^	33.3 ± 0.56 ^a^	28.8 ± 0.27 ^a^	44.1 ± 24.7 ^a^	26.6 ± 1.54 ^a^	30.5 ± 1.01 ^a^
met	14.6 ± 1.21^c^	34.9 ± 0.33 ^a^	20.7 ± 0.64 ^c^	30.9 ± 1.18 ^ab^	30.6 ± 1.59 ^ab^	19.4 ± 4.67 ^c^	13.4 ± 8.88 ^c^	21.7 ± 6.10 ^bc^	30.2 ± 0.78 ^ab^
phe	24.5 ± 9.31^a^	22.2 ± 0.11 ^a^	19.7 ± 6.13 ^a^	16.3 ± 4.03 ^a^	16.5 ± 2.57 ^a^	18.6 ± 4.58 ^a^	15.5 ± 5.14 ^a^	15.5 ± 4.60 ^a^	18.5 ± 1.81 ^a^
ile	17.2 ± 0.27 ^b^	20.2 ± 0.93 ^a^	16.9 ± 1.65 ^b^	14.3. ± 0.88 ^cd^	17.6 ± 0.23 ^b^	14.0 ± 0.97 ^d^	12.2 ± 0.95 ^d^	13.1 ± 1.28 ^d^	16.3 ± 0.19 ^bc^
leu	32.9 ± 1.05 ^de^	44.6 ± 0.33 ^a^	33.9 ± 0.46 ^cd^	38.6 ± 6.22 ^bc^	36.4 ± 0.17 ^bcd^	26.5 ± 0.14 ^f^	27.8 ± 1.45 ^ef^	28.6 ± 0.28 ^ef^	39.7 ± 0.77 ^ab^
lys	35.3 ± 1.06 ^a^	25.9 ± 0.27 ^bc^	34.2 ± 0.22 ^a^	23.3 ± 0.59 ^e^	21.6 ± 0.70 ^f^	24.6 ± 0.09 ^d^	26.6 ± 0.28 ^b^	18.8 ± 0.66 ^g^	25.2 ± 0.13 ^cd^
pro	18.3 ± 0.66 ^cd^	16.7 ± 0.52 ^d^	21.2 ± 0.27 ^abc^	19.6 ± 0.79 ^cd^	18.8 ± 2.67 ^cd^	23.1 ± 0.84 ^ab^	23.7 ± 2.28 ^a^	18.8 ± 0.21 ^cd^	20.5 ± 0.13 ^bc^
total	843 ± 6.86 ^c^	944 ± 13.1 ^b^	1021 ± 7.19 ^a^	800 ± 2.89 ^c^	910 ± 9.08 ^b^	914 ± 16.5 ^b^	833 ± 51.0 ^c^	845 ± 34.9 ^c^	910 ± 27.8 ^b^

* Different superscript letters in the same rows represent a significant difference (*p* < 0.05).

**Table 3:** Changes in OAV values of processed beef based on the addition of varying types of phosphate.

No.	Volatile Compounds	OT (μg/kg)	OAV								
			Control	SY	SYSP	SYST	SYSH	SP	ST	SH	SYCP
1	1-Octen-3-ol	1	82.66	100.21	5.30	1.44	86.71	-	2.28	6.19	2.21
2	Pentanal	12	25.54	16.44	1.72	0.43	13.70	-	-	1.27	-
3	Hexanal	4.5	202.17	116.87	24.45	3.47	98.63	3.12	5.90	19.12	4.12
4	Heptanal	3	49.66	64.38	8.05	1.74	43.23	1.32	2.83	3.61	1.72
5	Octanal	0.7	265.92	256.98	38.60	15.80	191.62	13.24	19.89	27.36	10.99
6	Nonanal	1	301.88	239.68	67.47	74.83	202.59	73.83	71.95	82.22	34.91
7	2-Octenal, (E)-	3	14.20	9.73	0.40	-	8.00	-	-	-	-
8	2,4-Heptadienal, (E,E)-	0.15	26.70	13.63	-	-	16.56	-	-	-	-
9	Decanal	0.1	304.79	258.25	78.66	112.95	265.15	105.07	118.26	101.36	64.91
10	Benzaldehyde	24	3.07	1.94	1.22	0.43	2.06	0.13	1.33	0.60	0.45
11	trans-2-Nonenal	0.19	118.28	77.82	3.63	-	63.94	-	-	-	-
12	cis-4-Decenal	0.04	290.48	198.77	-	-	170.17	-	-	-	-
13	2,6-Nonadienal, (E,Z)-	0.02	26.40	16.95	-	-	20.25	-	-	-	-
14	2-Decenal, (Z)-	0.4	29.62	21.71	1.48	-	17.23	-	-	-	-
15	2,4-Decadienal	0.3	46.76	26.36	-	-	23.78	-	-	-	-
16	2,4-Nonadienal, (E,E)-	0.09	120.02	63.81	-	-	44.61	-	-	-	-
17	2,3-Butanedione	0.059	3698.37	-	175.66	-	-	-	-	-	663.25
18	2-Decanone	1.43	1.21	1.15	-	-	1.66	-	-	-	-
19	3,5-Octadien-2-one, (E,E)-	0.15	56.47	52.21	-	-	-	-	-	-	-
20	2,3-Octanedione	2.52	-	113.24	5.54	-	103.76	-	0.37	5.07	0.40
21	D-Limonene	10	0.74	0.34	3.96	0.23	0.99	0.44	2.95	0.89	0.65
22	Disulfide, dimethyl	0.06	29.83	-	-	42.59	20.49	-	58.31	65.46	-
23	Furan, 2-pentyl-	6	14.42	15.35	-	0.12	16.08	0.09	0.30	0.98	0.23
24	Dimethyl trisulfide	0.005	2643.28	573.97	581.72	123.87	1073.82	-	1013.22	1413.13	111.20

**Table 4:** Values in sensory characteristics of prepared beef based on the addition of varying types of phosphate *.

Group	Appearance	Juiciness	Flavor	Tenderness	Overall Acceptability
control	5.11 ± 1.90 ^a^	3.67 ± 1.00 ^c^	3.44 ± 1.33 ^c^	4.11 ± 1.62 ^c^	3.22 ± 1.39 ^d^
SY	4.89 ± 1.36 ^a^	4.00 ± 0.87 ^c^	4.89 ± 1.27 ^b^	3.89 ± 1.62 ^c^	3.67 ± 1.32 ^cd^
SYSP	5.44 ± 1.74 ^a^	6.44 ± 1.01 ^a^	6.67 ± 1.41 ^a^	6.44 ± 1.94 ^ab^	6.78 ± 1.39 ^a^
SYST	5.00 ± 1.73 ^a^	6.44 ± 1.67 ^a^	6.00 ± 1.12 ^ab^	6.56 ± 1.94 ^a^	5.89 ± 1.69 ^ab^
SYSH	6.22 ± 1.39 ^a^	5.78 ± 1.56 ^ab^	7.00 ± 1.41 ^a^	6.00 ± 1.80 ^ab^	6.22 ± 1.64 ^ab^
SP	6.44 ± 1.33 ^a^	7.00 ± 1.58 ^a^	7.00 ± 1.66 ^a^	5.89 ± 1.17 ^ab^	6.78 ± 1.30 ^a^
ST	6.33 ± 1.80 ^a^	4.56 ± 1.42 ^bc^	5.89 ± 1.62 ^ab^	4.67 ± 1.80 ^bc^	5.00 ± 1.22 ^bc^
SH	5.89 ± 1.54 ^a^	5.56 ± 1.24 ^ab^	6.89 ± 1.45 ^a^	4.78 ± 1.79 ^abc^	5.89 ± 1.36 ^ab^
SYCP	5.11 ± 1.90 ^a^	6.56 ± 1.33 ^a^	5.78 ± 1.92 ^ab^	6.00 ± 1.66 ^ab^	5.78 ± 1.39 ^ab^

* Different superscript letters in the same column represent a significant difference (*p* < 0.05).

## Data Availability

The data presented in this study are available on request from the corresponding author.

## Supplementary Materials

The following supporting information can be downloaded at: https://www.mdpi.com/article/10.3390/molecules30010202/s1.
