# Supplementary material for: Influence of Phosphate Marinades on the Quality and Flavor Characteristics of Prepared Beef
Source: Molecules. 2025 Jan 6;30(1):202. doi: 10.3390/molecules30010202 (PMC11721332; doi:10.3390/molecules30010202)
Supplement: Supplementary file 1 [file molecules-30-00202-s001.zip › molecules-3340596-supplementary.pdf]

No.	Compounds	Concentration(μg/kg)[2]									Identification methods[3]
		control	SY	SYSP	SYST	SYSH	SP	ST	SH	SYCP	
V1	1-Pentanol	87.71±13.22[a]	44.20±5.00[b]	2.86±0.82[c]	-	38.31±10.49[b]	-	-	-	1.50±1.18[c]	RI,MS,S
V2	1-Hexanol	63.83±9.30[a]	32.92±2.77[b]	-	-	73.02±24.92[a]	-	-	8.61±0.59[c]	-	RI,MS,S
V3	1-Octen-3-ol	82.66±18.38[a]	100.21±0.89[a]	5.30±0.79[b]	1.44±0.07[d]	86.71±2.10[a]	-	2.28±0.36[c]	6.19±1.03[b]	2.21±0.29[c]	RI,MS,S
V4	2-Octen-1-ol, (E)-	6.41±2.12[a]	4.18±0.95[a]	-	-	-	-	-	-	-	RI,MS,S
V5	2-Tridecen-1-ol, (E)-	5.77±1.25[a]	3.03±0.70[b]	-	-	3.58±0.17[b]	-	1.64±0.32[c]	-	-	RI,MS
V6	Phenylethyl Alcohol	1.32±2.29[a]	-	0.25±0.01[a]	0.41±0.03[a]	0.21±0.03[a]	0.40±0.19[a]	0.37±0.09[a]	0.14±0.02[a]	0.22±0.06[a]	RI,MS
V7	1-Hexanol, 2-ethyl-	0.29±0.01[c]	-	3.10±0.90[b]	5.76±2.47[a]	3.24±0.32[b]	-	5.47±1.25[ab]	4.19±0.74[b]	6.60±0.79[a]	RI,MS
V8	1-Octanol	31.30±8.64[a]	23.57±0.14[b]	2.38±0.58[d]	-	14.38±0.47[c]	2.37±0.36[d]	1.94±0.18[e]	2.59±0.11[d]	-	RI,MS,S
V9	Ethanol, 2-(2-butoxyethoxy)-	-	1.11±0.11[d]	-	-	1.45±0.10[b]	-	1.24±0.04[c]	1.03±0.18[d]	1.99±0.15[a]	RI,MS
V10	Benzenemethanol, α,α-dimethyl-	-	-	0.47±0.07[a]	0.32±0.01[b]	-	-	0.22±0.01[c]	-	0.45±0.02[a]	RI,MS
V11	Butanal	15.86±3.75[a]	11.78±6.18[b]	-	-	1.53±0.94[c]	-	-	-	-	RI,MS
V12	Pentanal	306.43±43.82[a]	197.34±7.74[b]	20.68±2.24[c]	5.11±0.15[d]	164.43±9.89[b]	-	-	15.20±5.23[c]	-	RI,MS,S
V13	Hexanal	909.78±115.86[a]	525.90±64.62[b]	110.02±10.06[c]	15.61±0.68[e]	443.83±7.51[b]	14.04±4.62[e]	26.54±3.33[e]	86.03±4.57[d]	18.54±8.01[e]	RI,MS,S
V14	Heptanal	148.97±21.29[a]	193.14±34.79[a]	24.14±1.57[b]	5.22±1.37[d]	129.69±25.40[a]	3.97±1.97[d]	8.48±0.80[c]	10.83±0.22[c]	5.17±0.22[d]	RI,MS,S
V15	2-Hexenal, (E)-	12.58±5.55[a]	-	-	-	-	-	-	-	-	RI,MS,S
V16	Octanal	186.15±22.93[a]	179.89±7.66[b]	27.02±1.97[d]	11.06±1.16[g]	134.13±9.10[c]	9.27±1.76[g]	13.92±1.09[f]	19.15±0.26[e]	7.69±3.51[g]	RI,MS,S

V17	2-Hexenal, 2-ethyl-	36.62±8.72^{a}	-	-	-	-	-	-	-	-	RI,MS
V18	Nonanal	301.88±34.29^{a}	239.68±2.78^{b}	67.47±3.82^{c}	74.83±9.24^{c}	202.59±13.27^{b}	73.83±15.58^{c}	71.95±1.47^{c}	82.22±21.89^{c}	34.91±7.48^{d}	RI,MS,S
V19	5-Ethylcyclopent-1-enecarboxaldehyde	11.81±1.77^{a}	7.08±0.74^{b}	-	-	-	-	-	-	-	RI,MS
V20	2-Octenal, (E)-	42.61±7.33^{a}	29.19±0.04^{c}	1.21±0.25^{d}	-	23.99±1.07^{c}	-	-	-	-	RI,MS,S
V21	2,4-Heptadienal, (E,E)-	4.00±0.51^{a}	2.04±0.31^{b}	-	-	2.48±1.00^{b}	-	-	-	-	RI,MS,S
V22	Decanal	30.48±17.34^{a}	25.83±12.69^{a}	7.87±3.97^{b}	11.30±0.79^{b}	26.52±0.86^{a}	10.51±2.14^{b}	11.83±1.56^{b}	10.14±4.09^{b}	6.49±3.47^{b}	RI,MS
V23	Benzaldehyde	73.76±7.34^{a}	46.59±0.77^{b}	29.39±3.01^{c}	10.42±0.64^{d}	49.46±2.60^{b}	3.12±0.54	32.04±1.96^{c}	14.47±1.63^{d}	10.74±1.72^{d}	RI,MS,S
V24	trans-2-Nonenal	22.47±4.17^{a}	14.79±0.10^{b}	0.69±0.16	-	12.15±1.10^{c}	-	-	-	-	RI,MS,S
V25	cis-4-Decenal	11.62±2.60^{a}	7.95±0.10^{b}	-	-	6.81±0.40^{c}	-	-	-	-	RI,MS
V26	2,6-Nonadienal, (E,Z)-	0.53±0.19^{a}	0.34±0.01^{a}	-	-	0.41±0.02^{a}	-	-	-	-	RI,MS
V27	2,4-Octadienal, (E,E)-	1.82±0.35^{a}	1.32±0.01^{b}	-	-	1.19±0.05^{c}	-	-	-	-	RI,MS
V28	Benzeneacetaldehyde	0.67±0.19^{a}	0.41±0.58^{b}	0.40±0.01^{b}	-	0.56±0.03^{ab}	-	0.48±0.05^{b}	-	0.63±0.19^{a}	RI,MS
V29	2-Decenal, (Z)-	11.85±2.24^{a}	8.68±0.24^{b}	-	-	6.89±0.30^{c}	-	-	-	-	RI,MS,S
V30	2-Octenal, 2-butyl-	7.24±1.37^{a}	4.64±0.36^{b}	-	-	-	-	-	-	-	RI,MS
V31	Benzaldehyde, 4-ethyl-	2.96±0.66^{b}	2.19±0.17^{b}	0.88±1.01^{c}	0.18±0.08^{d}	5.00±6.09^{a}	-	0.21±0.07^{d}	0.19±0.07^{d}	-	RI,MS

V32	Dodecanal	16.21±3.11^{a}	12.05±0.76^{b}	1.72±0.30^{c}	1.37±0.13^{c}	10.42±1.42^{b}	0.93±0.06^{d}	1.38±0.18^{c}	1.52±0.13^{c}	1.17±0.04^{c}	RI,MS
V33	2-Thiophenecarboxaldehyde	0.14±0.02^{c}	0.33±0.01^{b}	0.51±0.43^{a}	0.22±0.04^{c}	0.38±0.02^{b}	-	-	-	0.54±0.36^{a}	RI,MS
V34	Benzaldehyde, 4-(1-methylethyl)-	0.25±0.01^{b}	0.20±0.01^{c}	0.88±1.01^{a}	0.24±0.27	0.16±0.02^{d}	0.21±0.03^{c}	0.20±0.04^{c}	0.27±0.03^{b}	-	RI,MS
V35	2,4-Decadienal, (E,Z)-	14.03±2.25^{a}	7.91±0.38^{b}	-	-	7.13±1.02^{b}	-	-	-	-	RI,MS,S
V36	Tridecanal	31.29±5.98^{d}	23.14±2.09^{b}	1.72±0.46^{c}	0.57±0.07^{d}	19.81±2.15^{b}	0.47±0.04^{d}	0.78±0.05^{d}	1.03±0.15^{cd}	0.49±0.02^{d}	RI,MS
V37	Benzaldehyde, 4-pentyl-	1.12±0.21^{b}	1.27±0.10^{a}	-	-	1.11±0.11^{b}	-	-	-	-	RI,MS
V38	Pentadecanal-	55.26±10.94^{a}	42.36±4.67^{b}	5.06±1.59^{c}	1.95±0.24^{d}	49.33±5.58^{b}	1.33±0.41^{d}	4.66±0.14^{c}	3.40±0.37^{cd}	2.00±0.15^{d}	RI,MS
V39	Cinnamaldehyde, (E)-	2.19±0.51^{a}	-	-	-	1.61±0.10^{b}	-	-	-	-	RI,MS
V40	Hexadecanal	68.37±6.37^{a}	34.16±5.55^{b}	37.60±9.85^{b}	17.33±2.52^{c}	71.78±13.42^{a}	6.74±2.21^{d}	51.55±3.56^{a}	37.10±2.35^{b}	16.95±2.18^{c}	RI,MS
V41	cis-9-Hexadecenal	1.48±0.17^{b}	1.12±0.07^{b}	0.60±0.09^{d}	0.30±0.01^{d}	2.19±0.38^{a}	-	0.93±0.15^{b}	0.66±0.04^{c}	-	RI,MS
V42	Heptadecanal	7.84±1.19^{a}	5.08±0.66^{b}	1.38±0.33^{c}	0.78±0.06^{d}	7.01±0.69^{a}	0.72±0.22^{d}	1.57±0.09^{c}	0.98±0.06^{c}	0.70±0.05^{d}	RI,MS
V43	13-Octadecenal, (Z)-	1.17±0.42^{a}	0.81±0.19^{b}	-	-	1.70±0.32^{a}	-	-	-	-	RI,MS
V44	4,8,12-Tetradecatrienal, 5,9,13-trimethyl-	2.52±0.37^{a}	-	0.46±0.19^{b}	0.33±0.03^{c}	0.67±0.19^{b}	0.31±0.06^{c}	0.42±0.13^{b}	-	-	RI,MS
V45	Octadecanal	6.18±0.53^{a}	2.32±0.92^{bc}	3.68±1.00^{b}	1.38±0.28^{c}	6.41±1.96^{a}	0.81±0.17^{d}	4.37±0.75^{b}	2.35±0.46^{bc}	1.40±0.31^{c}	RI,MS
V46	9-Octadecenal	0.77±0.03^{d}	3.34±2.11^{a}	0.60±0.16^{d}	0.42±0.08^{d}	2.41±0.27^{b}	-	0.93±0.10^{c}	0.79±0.13^{d}	-	RI,MS
V47	U-ecanal	8.84±2.19^{a}	6.18±0.41^{b}	-	1.38±0.09^{c}	5.26±0.50^{b}	0.87±0.03^{c}	-	0.37±0.03^{d}	-	RI,MS,S

V48	2,4-Nonadienal, (E,E)-	10.80±1.81[a]	5.74±0.38[b]	-	-	4.01±0.94[b]	-	-	-	-	RI,MS
V49	Furfural	-	-	0.80±0.13[b]	0.22±0.01[c]	-	0.18±0.04[c]	-	-	3.50±0.06[a]	RI,MS
V50	Benzaldehyde, 3,4-dimethyl-	-	-	0.21±0.07[a]	0.11±0.01[b]	-	0.11±0.01[b]	0.08±0.01[c]	-	-	RI,MS
V51	Benzaldehyde, 2,4,5-trimethyl-	-	-	0.97±0.23[a]	-	-	0.28±0.03[b]	-	-	-	RI,MS
V52	2,5-Fura-icarboxaldehyde	-	-	0.80±0.13[b]	0.82±0.08[b]	-	0.60±0.03[b]	-	0.75±0.08[b]	2.37±0.78[a]	RI,MS
V53	Benzaldehyde, 2-methyl-	-	-	-	-	2.21±2.78[a]	0.31±0.02[b]	0.06±0.05[c]	0.06±0.01[c]	-	RI,MS
V54	Benzaldehyde, 4-methoxy-	-	-	-	0.11±0.11[b]	-	-	0.06±0.03[b]	0.45±0.05[a]	0.45	RI,MS
V55	Butanal, 3-methyl-	-	-	-	-	2.80±0.59[b]	0.27±0.04[d]	1.28±0.70[c]	0.60±0.18[d]	9.39±1.70[a]	RI,MS
V56	2,3-Butanedione	218.20±35.01[a]	-	10.36±12.51[c]	-	-	-	-	-	39.13±4.33[b]	RI,MS
V57	2,3-Pentanedione	9.54±1.07[a]	5.77±0.05[c]	-	-	6.22±0.54[b]	-	-	-	-	RI,MS
V58	2-Heptanone, 6-methyl-	5.81±1.90[b]	-	-	-	7.32±5.03[a]	-	-	-	-	RI,MS
V59	3-Octen-2-one	5.16±1.43[a]	1.34±1.89[b]	-	-	-	-	-	-	-	RI,MS,S
V60	2-Propanone, 1-(acetyloxy)-	9.57±0.88[a]	-	1.15±0.60[c]	0.52±0.08[d]	4.66±0.98[b]	0.78±0.20[c]	0.82±0.08[c]	0.74±0.18[c]	0.99±0.26[c]	RI,MS
V61	2-Decanone	1.73±0.28[ab]	1.64±0.16[b]	-	-	2.37±1.59[a]	-	-	-	-	RI,MS
V62	3,5-Octadien-2-one	1.71±0.41[a]	1.15±0.04[b]	-	-	0.70±0.07[c]	-	-	-	-	RI,MS

V63	3,5-Octadien-2-one, (E,E)-	8.47±2.98^{a}	7.83±0.44^{a}	-	-	-	-	-	-	-	RI,MS
V64	Acetophenone	0.31±0.11^{c}	0.56±0.07^{b}	0.46±0.03^{bc}	0.50±0.12^{b}	0.53±0.09^{b}	0.25±0.03^{c}	0.33±0.04^{c}	0.41±0.04^{bc}	0.71±0.08^{a}	RI,MS
V65	2(3H)-Furanone, 5-butyldihydro-	0.38±0.04^{a}	0.29±0.00^{a}	0.29±0.06^{a}	0.16±0.13	-	0.25±0.10^{a}	0.17±0.08^{b}	0.19±0.00^{b}	0.24±0.03^{a}	RI,MS,S
V66	2-Butanone	6.84±1.45^{a}	1.96±2.77^{b}	1.18±0.52^{b}	-	-	-	-	3.73±0.47^{b}	0.67±0.18^{c}	RI,MS
V67	2,3-Nonanedione	2.44±0.65^{a}	-	-	-	-	-	-	-	-	RI,MS
V68	2-U-ecanone	0.55±0.04^{a}	-	0.39±0.07^{b}	0.32±0.05^{b}	-	-	-	-	0.29±0.18^{b}	RI,MS
V69	5,9-U-ecadien-2-one, 6,10-dimethyl-, (E)-	2.22±0.15^{a}	1.00±0.04^{b}	0.57±0.08^{bc}	0.52±0.08^{c}	0.87±0.10^{b}	0.32±0.00^{c}	0.45±0.05^{c}	0.36±0.05^{c}	0.51±0.00^{c}	RI,MS
V70	2,3-Octanedione	-	285.37±5.92^{a}	13.96±2.66^{c}	-	261.47±7.53^{b}	-	0.93±0.19^{d}	12.79±1.68^{c}	1.01±0.18^{d}	RI,MS
V71	2-Heptanone	-	124.94±0.88^{a}	-	-	-	-	-	-	-	RI,MS
V72	5-Hepten-2-one, 6-methyl-	-	-	1.27±0.08^{b}	1.74±0.27^{a}	-	1.09±0.12^{b}	1.73±0.27^{a}	1.48±0.14^{b}	1.90±0.41^{a}	RI,MS,S
V73	2-Nonanone	-	-	0.33±0.14^{b}	0.48±0.09^{b}	-	0.44±0.02^{b}	0.65±0.10^{a}	0.39±0.14^{b}	-	RI,MS
V74	Ethanone, 1-(4,5-dihydro-2-thiazolyl)-	-	-	0.25±0.06^{c}	0.17±0.03^{c}	-	0.46±0.05^{b}	0.14±0.01^{c}	-	0.71±0.05^{a}	RI,MS
V75	3-Heptanone	-	-	2.44±0.10^{a}	0.98±1.30^{b}	2.09±0.46^{a}	0.15±0.04^{b}	-	0.13±0.01^{b}	-	RI,MS
V76	2-Tridecanone	-	-	0.39±0.07^{a}	0.32±0.05^{a}	-	0.20±0.02^{b}	-	-	-	RI,MS
V77	2-Pentadecanone	-	-	-	0.48±0.02^{a}	-	0.52±0.06^{a}	-	-	0.29±0.18^{b}	RI,MS
V78	n-Caproic acid vinyl ester	3.31±1.30^{b}	1.27±0.37^{c}	-	-	129.97±0.52^{a}	-	-	-	-	RI,MS
V79	Dodecanoic acid, methyl ester	1.00±0.41^{b}	-	-	-	0.29±0.06^{c}	-	-	1.23±0.31^{a}	0.71±0.21^{b}	RI,MS

V80	Hexadecanoic acid, methyl ester	1.02±0.27^{a}	0.32±0.01^{b}	0.21±0.03^{c}	0.19±0.02^{c}	0.38±0.03^{b}	0.24±0.04^{c}	0.35±0.04^{b}	-	0.22±0.01^{c}	RI,MS
V81	Hexadecanoic acid, ethyl ester	0.36±0.23^{b}	0.56±0.08^{a}	0.24±0.06^{c}	0.22±0.01^{c}	0.19±0.02^{d}	0.21±0.06^{c}	0.22±0.10^{c}	-	-	RI,MS
V82	Dimethyl phthalate	0.48±0.18^{a}	0.18±0.01^{c}	-	-	0.35±0.05^{b}	-	-	-	0.14±0.03^{c}	RI,MS
V83	δ-Dodecalactone	0.63±0.16^{b}	0.36±0.01^{c}	0.26±0.04^{d}	0.24±0.04^{d}	3.83±1.07^{a}	0.31±0.01^{c}	0.25±0.04^{d}	0.35±0.06^{c}	0.47±0.00^{c}	RI,MS
V84	1,2-Benzenedicarboxylic acid, bis(2-methylpropyl) ester	5.32±2.12^{a}	3.71±0.92^{b}	2.53±0.27^{c}	3.13±0.18^{b}	0.31±0.04^{b}	2.67±0.38^{b}	3.31±0.59^{b}	3.23±0.79^{b}	1.20±0.18^{d}	RI,MS
V85	γ-Tetradecalactone	0.70±0.17^{b}	0.41±0.02^{c}	0.26±0.04^{d}	0.18±0.02^{d}	3.12±2.35^{a}	0.20±0.01^{d}	-	-	-	RI,MS
V86	1,2-Benzenedicarboxylic acid, butyl 2-ethylhexyl ester	3.77±0.62^{ab}	7.22±2.98^{a}	2.23±1.76^{b}	3.12±2.43^{ab}	-	3.88±0.91^{ab}	3.48±0.08^{ab}	3.91±1.51^{ab}	0.66±0.23^{c}	RI,MS
V87	1-Propen-2-ol, acetate	-	42.64±0.36^{a}	31.07±6.39^{b}	-	-	-	-	-	-	RI,MS
V88	Butyrolactone	-	0.69±0.07^{b}	0.76±0.08^{b}	0.80±0.03^{b}	1.20±0.18^{a}	0.30±0.02^{c}	0.72±0.03^{b}	0.87±0.08^{b}	1.00±0.14^{a}	RI,MS,S
V89	γ-Dodecalactone	-	0.87±1.23^{b}	1.18±0.16^{a}	0.81±0.08^{b}	1.78±0.23^{a}	0.77±0.14^{b}	-	0.99±0.13^{b}	0.81±0.12^{b}	RI,MS
V90	2,2,4-Trimethyl-1,3-pentanediol diisobutyrate	-	-	-	0.27±0.02^{a}	-	0.15±0.02^{b}	-	0.13±0.00^{b}	-	RI,MS

V91	Carbamodithioic acid, diethyl-, methyl ester	-	-	-	0.48±0.02[ab]	-	0.52±0.06[a]	0.68±0.07[a]	-	0.39±0.04[b]	RI,MS
V92	Sulfurous acid, dodecyl pentyl ester	-	-	-	-	-	-	0.42±0.04[a]	-	0.43±0.16[a]	RI,MS
V93	Propanoic acid	1.94±0.69[a]	-	1.55±0.76[ab]	-	1.26±0.33[b]	-	1.56±0.20[ab]	-	-	RI,MS
V94	Decane	68.10±12.18[a]	37.05±3.52[bc]	27.15±6.33[c]	10.34±1.36[d]	44.93±6.39[b]	-	-	22.06±3.31[c]	27.43±0.65[c]	RI,MS
V95	Nonane, 3-methylene-	6.96±1.29[a]	-	7.57±1.12[a]	1.95±0.15[c]	6.10±0.75[a]	-	4.12±0.21[b]	3.55±1.25[b]	-	RI,MS
V96	Decane, 5-methyl-	0.25±0.23[ab]	0.18±0.01[b]	0.19±0.08[b]	-	0.35±0.08[a]	-	0.15±0.03[b]	0.27±0.06[a]	0.32±0.05[a]	RI,MS
V97	Decane, 3-methyl-	0.51±0.13[a]	-	0.20±0.08[c]	-	0.46±0.07[a]	-	0.18±0.02[c]	0.23±0.05[b]	0.29±0.08[b]	RI,MS
V98	U-ecane	74.14±1.99[a]	55.33±9.68[b]	50.43±6.23[b]	20.03±2.97[d]	62.92±6.94[b]	19.90±4.30	36.62±2.48[c]	59.60±8.80[b]	68.51±12.12[a]	RI,MS
V99	5-Ethyldecane	13.44±5.76[a]	4.09±3.51[b]	1.74±1.07[c]	0.22±0.12[d]	4.89±0.02[b]	0.69±0.82[d]	1.60±0.96[c]	1.24±0.25[c]	3.72±1.34[b]	RI,MS
V100	U-ecane, 2-methyl-	1.93±2.74[a]	1.47±0.01[a]	0.49±0.06[b]	-	-	-	-	1.13±1.01[a]	0.35±0.23[b]	RI,MS
V101	U-ecane, 3-methyl-	17.93±1.07[a]	4.92±2.96[c]	9.20±2.05[b]	-	14.25±3.85[a]	-	5.08±1.79[c]	-	8.56±0.41[b]	RI,MS
V102	Dodecane	160.77±7.87[a]	90.05±14.91[c]	64.76±12.52[d]	30.81±1.49[e]	123.78±12.63[b]	31.15±11.64[e]	67.84±2.81[d]	74.21±13.80[cd]	81.54±17.75[c]	RI,MS
V103	Tridecane	72.18±0.88[a]	34.83±3.00[b]	25.49±10.56[b]	12.71±1.67[c]	64.06±9.24[a]	12.41±5.73[c]	24.09±2.14[b]	31.99±12.77[b]	30.45±10.36[b]	RI,MS
V104	Tridecane, 3-methyl-	7.52±1.26[a]	5.97±0.09[b]	2.29±1.79[c]	2.74±0.28[c]	6.35±1.44[ab]	1.97±0.18[c]	3.22±0.45[c]	3.64±3.01[bc]	3.10±1.38[c]	RI,MS
V105	Pentadecane	20.69±2.28[a]	9.94±0.38[b]	-	-	7.57±0.04[c]	2.82±1.48[d]	3.21±0.19[d]	4.15±1.25[d]	3.47±1.73[d]	RI,MS

V106	Hexadecane	6.30±1.44^{a}	4.62±0.79^{b}	2.01±0.12^{d}	2.80±0.46	3.36±0.07^{c}	2.06±0.45^{d}	2.16±0.19^{d}	3.01±0.98^{c}	2.38±0.05^{d}	RI,MS
V107	13-Methyltetradecanal	6.78±2.51^{a}	3.68±0.18^{b}	0.55±0.01^{e}	2.05±0.03^{c}	3.97±1.02^{ab}	0.89±0.12^{e}	0.73±0.08^{e}	1.16±0.08^{d}	1.79±0.12^{c}	RI,MS
V108	Octadecane	2.06±1.00^{a}	1.14±0.14^{b}	0.74±0.08^{c}	0.79±0.13^{c}	0.79±0.18^{c}	0.94±0.23^{c}	0.80±0.15^{c}	0.50±0.04^{d}	0.76±0.09^{c}	RI,MS
V109	Tetradecanal	43.22±11.72^{a}	33.16±3.86^{a}	8.32±4.40^{c}	12.85±1.11^{b}	39.59±17.80^{a}	11.61±1.98^{c}	12.85±2.53^{b}	16.19±0.32^{b}	9.48±1.80^{c}	RI,MS
V110	Dodecane, 4-methyl-	4.01±0.29^{a}	0.81±0.14^{c}	0.58±0.28^{c}	-	1.67±0.86^{b}	-	1.25±1.68^{b}	-	-	RI,MS
V111	U-ecane, 3-methylene-	7.86±0.54^{a}	9.58±3.54^{a}	-	0.93±0.02^{c}	-	0.60±0.16^{c}	2.90±0.47^{b}	2.15±0.96^{b}	1.82±0.57^{b}	RI,MS
V112	Tetradecane, 3-methyl-	0.39±0.08^{a}	-	-	-	-	-	-	0.20±0.01^{b}	-	RI,MS
V113	Heptadecane	10.80±1.81^{a}	-	1.11±0.18^{c}	0.75±0.07^{d}	4.06±1.01^{b}	0.81±0.15^{d}	0.73±0.08^{d}	1.08±0.19^{c}	1.08±0.05^{c}	RI,MS
V114	Docosane	-	0.33±0.09^{a}	0.16±0.01^{c}	-	0.25±0.12^{a}	0.20±0.04^{b}	0.27±0.14^{a}	0.26±0.11^{a}	-	RI,MS
V115	Tetracosane	-	0.89±0.13^{a}	-	0.26±0.20^{b}	-	0.13±0.00^{d}	0.18±0.07^{c}	-	-	RI,MS
V116	Hexadecane, 2,6,10,14-tetramethyl-	-	-	0.45±0.07^{b}	0.54±0.07^{ab}	0.59±0.01^{a}	0.43±0.05^{b}	0.61±0.07^{a}	-	0.39±0.04^{b}	RI,MS
V117	Tridecane, 3-methylene-	-	-	1.34±0.12^{a}	-	-	-	1.81±0.17^{a}	-	-	RI,MS
V118	Octane, 2,3-dimethyl-	-	-	-	1.07±0.29^{a}	-	1.03±0.62^{a}	-	-	-	RI,MS
V119	Octadecane, 6-methyl-	-	-	-	0.35±0.06^{a}	-	-	0.53±0.27^{a}	-	-	RI,MS
V120	Pentadecane, 3-methyl-	-	-	1.07±0.57^{a}	1.55±0.24^{a}	-	0.72±0.14^{b}	1.45±0.17^{a}	1.39±0.33^{a}	1.22±0.31^{a}	RI,MS

V121	Cyclohexane	-	-	-	9.17±0.94^{a}	-	1.26±0.15^{c}	0.11±0.08^{d}	0.21±0.01^{d}	3.37±1.00^{b}	RI,MS
V122	Oxirane, tetradecyl-	-	-	-	0.78±0.12^{c}	-	0.34±0.04^{d}	2.91±0.02^{a}	0.94±0.64^{bc}	1.57±0.14^{b}	RI,MS
V123	2-Ethyl-oxetane	-	-	-	-	-	-	1.50±0.42^{b}	27.01±19.01^{a}	25.45±12.84^{a}	RI,MS
V124	Ethylbenzene	6.74±3.16^{a}	1.03±0.02^{c}	1.10±0.17^{bc}	1.04±0.23	2.21±1.22^{b}	0.66±0.17^{c}	0.68±0.32^{c}	1.17±0.33^{bc}	1.30±0.55^{bc}	RI,MS
V125	p-Xylene	12.89±6.15^{a}	-	1.30±1.13^{c}	0.92±0.37^{c}	8.08±5.42^{a}	0.79±0.58^{c}	-	1.04±0.04^{c}	4.38±2.06^{b}	RI,MS
V126	o-Xylene	0.97±0.90^{ab}	-	-	1.43±0.49^{a}	1.09±0.23^{ab}	0.76±0.39^{b}	2.14±0.14^{a}	-	1.14±0.52^{a}	RI,MS
V127	Benzene, 1,2,4-trimethyl-	0.24±0.13^{b}	-	0.80±0.56^{a}	-	-	-	-	-	0.33±0.06^{b}	RI,MS
V128	2,4-Di-tert-butylphenol	3.91±2.06^{a}	1.46±0.32^{ab}	0.81±0.18^{b}	0.92±0.23	1.35±0.17^{ab}	0.67±0.04^{c}	0.90±0.26^{b}	0.73±0.10^{b}	1.14±0.28^{ab}	RI,MS
V129	Benzene, 1,3-dimethyl-	13.70±5.47^{a}	0.59±0.20^{d}	0.57±0.02^{d}	-	7.01±2.38^{b}	-	3.02±0.99^{c}	0.57±0.00^{d}	2.08±1.82^{c}	RI,MS
V130	Toluene	-	7.27±0.32^{b}	7.57±1.12^{b}	1.95±0.15^{d}	2.75±2.36^{c}	1.75±0.25^{d}	4.12±0.21^{c}	3.89±0.59^{c}	14.72±1.44^{a}	RI,MS
V131	Benzene, 1,3-bis(1,1-dimethylethyl)-	-	9.67±1.86^{b}	-	-	11.72±0.23^{a}	-	-	-	1.41±0.24^{c}	RI,MS
V132	Mesitylene	-	-	1.16±0.12^{a}	0.30±0.27^{c}	0.48±0.40^{b}	0.81±0.99^{b}	-	-	-	RI,MS
V133	Benzene, 1-methyl-3-(1-methylethyl)-	-	-	0.36±0.08^{c}	0.69±0.09^{b}	2.10±1.95^{a}	-	1.65±1.06^{a}	1.12±0.70^{a}	0.74±0.12^{b}	RI,MS
V134	Benzene, 1,2,3,4-tetramethyl-	-	-	0.80±0.32^{b}	0.18±0.02^{c}	-	-	0.25±0.05^{c}	0.28±0.07^{c}	1.84±0.13^{a}	RI,MS
V135	Benzene, 1-ethyl-3-methyl-	-	-	-	0.39±0.46^{b}	-	-	0.52±0.12^{a}	-	0.37±0.13^{b}	RI,MS

V136	Butylated Hydroxytoluene	-	-	-	0.39±0.03^{a}	-	-	0.33±0.08^{a}	-	-	RI,MS
V137	Benzene, 1-ethyl-2,4-dimethyl-	-	-	-	-	-	-	-	-	0.44±0.17^{a}	RI,MS
V138	1-Nonene	0.19±0.04^{b}	-	-	-	0.34±0.24^{a}	-	-	-	-	RI,MS
V139	1,3-Octadiene	3.96±0.72^{a}	1.97±2.78^{b}	-	-	-	-	-	-	-	RI,MS
V140	D-Limonene	7.38±1.49^{c}	3.41±1.37^{d}	39.6±3.24^{a}	2.34±2.02^{d}	9.87±5.90^{c}	4.36±0.87^{d}	29.4±0.25^{b}	8.91±2.14^{c}	6.48±3.62^{c}	RI,MS,S
V141	1-Tridecene	3.92±0.29^{a}	4.86±1.01^{a}	-	-	2.55±0.08^{b}	-	1.35±0.21^{b}	1.16±1.21^{b}	3.66±0.59^{a}	RI,MS
V142	(3E,5Z)-1,3,5-U-ecatriene	0.36±0.20^{c}	1.34±1.89^{a}	-	-	0.70±0.01^{b}	-	-	-	-	RI,MS
V143	1-Pentadecene	2.65±0.86^{a}	2.75±0.04^{a}	0.57±0.04^{b}	-	2.16±0.10^{a}	-	0.14±0.04^{c}	-	-	RI,MS
V144	2-Hexadecene, 3,7,11,15-tetramethyl-, [R-[R*,R*-(E)]]-	0.87±0.86^{a}	-	0.84±0.19^{a}	0.61±0.02^{b}	0.25±0.04^{c}	0.91±0.10^{a}	0.82±0.03^{a}	-	0.86±0.09^{a}	RI,MS
V145	1-Decene	-	2.79±0.01^{b}	1.47±0.26^{c}	-	3.22±0.39^{a}	-	0.97±0.10^{c}	-	-	RI,MS
V146	3-U-ecene, 9-methyl-, (E)-	-	-	5.78±1.29^{a}	-	-	-	4.52±0.65^{a}	-	-	RI,MS
V147	Styrene	-	-	6.48±1.87^{a}	-	-	-	-	-	-	RI,MS
V148	5-Ethyl-1-nonene	-	-	0.61±0.16^{a}	-	-	-	0.57±0.06^{a}	-	0.45±0.17^{a}	RI,MS
V149	Caryophyllene	-	-	0.89±0.07^{a}	-	-	0.77±0.07^{a}	-	-	-	RI,MS,S
V150	1,3,5,7-Cyclooctatetraene	-	-	-	-	-	-	7.24±2.25^{a}	8.96±2.65^{a}	5.97±0.74^{a}	RI,MS
V151	Disulfide, dimethyl	1.79±1.99^{b}	-	-	2.56±2.49^{ab}	1.23±0.39^{b}	-	3.50±1.81^{a}	3.93±1.46^{a}	-	RI,MS
V152	2-n-Butyl furan	14.52±5.43^{a}	6.66±3.75^{b}	-	-	-	-	-	-	-	RI,MS,S

V153	Furan, 2-pentyl-	86.55±14.04[a]	92.11±13.46[a]	-	0.74±0.06[d]	96.50±3.96[a]	0.53±0.15	1.78±0.24[c]	5.86±1.83[b]	1.36±0.12[c]	RI,MS,S
V154	Mesitylene	15.34±3.85[b]	1.10±0.56[d]	-	1.24±0.32	0.61±0.01[d]	0.73±0.15[d]	5.88±1.99[c]	3.87±0.91[c]	23.72±5.30[a]	RI,MS,S
V155	Dimethyl trisulfide	13.22±5.47[a]	2.87±0.45[d]	2.91±0.98[d]	0.62±0.06[e]	5.37±2.26[bc]	-	5.07±0.37[c]	7.07±0.55[b]	0.56±0.15[e]	RI,MS
V156	Naphthalene	4.14±0.57[a]	3.64±0.12[b]	0.98±0.02[c]	1.50±0.25[c]	-	-	1.50±0.10[c]	1.71±0.03[c]	4.02±0.08[a]	RI,MS
V157	Anethole	0.62±0.16[b]	0.61±0.11[b]	0.55±0.10[b]	1.55±0.80[ab]	1.42±1.46[ab]	1.29±0.20[ab]	2.48±1.04[a]	2.14±0.21[a]	0.91±0.29[b]	RI,MS
V158	Naphthalene, 1-methyl-	0.18±0.17[b]	0.28±0.00[a]	0.13±0.02[b]	0.21±0.10[a]	0.19±0.02[b]	0.12±0.01[b]	0.23±0.02[a]	0.24±0.03[a]	0.57±0.01[a]	RI,MS
V159	p-Cresol	0.55±0.17[a]	0.41±0.00[a]	0.19±0.03[b]	0.17±0.01[b]	0.61±0.10[a]	0.16±0.01[b]	0.19±0.08[b]	0.18±0.03[b]	-	RI,MS
V160	2-Acetylthiazole	-	-	0.62±0.04[a]	0.44±0.06[b]	-	0.32±0.01[c]	0.52±0.02[ab]	-	1.27±0.12[a]	RI,MS
V161	estragole	-	-	1.66±0.41[b]	2.82±0.39[a]	-	2.69±0.21[a]	3.79±1.06[a]	3.01±0.25[a]	2.09±0.07[b]	RI,MS
V162	Dimethyl sulfone	-	-	-	0.19±0.04[b]	-	0.14±0.04[b]	0.19±0.01[b]	-	0.33±0.01[a]	RI,MS
V163	Benzothiazole	-	-	-	-	-	0.48±0.01[b]	0.48±0.06[b]	0.60±0.05[a]	-	RI,MS,S
V164	Diallyl disulphide	-	-	-	-	-	-	0.25±0.15[a]	0.31±0.13[a]	-	RI,MS

[2]Means ± standard derivations (n = 3); means within the same row with different letters indicated significant differences ($p < 0.05$); dash symbols (-) indicated the compounds were undetected, for which no signals were observed or the observed signals were less than 3 times the background signals. [3]RI, identified by comparison with the retention indices published in literature; MS, identified by search of the NIST 17 and 17s mass spectral database; S, identified by injection of available authentic chemicals.
